# Immediate Effect of Rigid Taping and Patella-Stabilizing Brace on Proprioception, Functionality, and Balance in Patients with Patellofemoral Pain Syndrome: A Randomised Controlled Trial

**DOI:** 10.3390/jcm15051936

**Published:** 2026-03-04

**Authors:** Ömer Naci Ergin, Ayşenur Erekdağ, İrem Nur Şener, Pelin Vural, Yıldız Analay Akbaba

**Affiliations:** 1 Department of Orthopaedics and Traumatology, Istanbul Faculty of Medicine, Istanbul University, Istanbul 34093, Türkiye; omer.ergin@istanbul.edu.tr; 2 Department of Physiotherapy and Rehabilitation, Institute of Graduate Studies, Istanbul University-Cerrahpasa, Istanbul 34320, Türkiye; aysenurerekdag@gmail.com (A.E.); iremnursener@aydin.edu.tr (İ.N.Ş.); pelin.vural@medipol.edu.tr (P.V.); 3 Division of Physiotherapy and Rehabilitation, Faculty of Health Sciences, Bezmialem Vakif University, Istanbul 34065, Türkiye; 4 Physiotherapy Program, Vocational School of Health Services, Istanbul Aydin University, Istanbul 34295, Türkiye; 5 Division of Physiotherapy and Rehabilitation, Faculty of Health Sciences, Istanbul Medipol University, Istanbul 34810, Türkiye; 6 Division of Physiotherapy and Rehabilitation, Faculty of Health Sciences, Istanbul University-Cerrahpasa, Istanbul 34500, Türkiye

**Keywords:** patellofemoral pain syndrome, function, brace, taping, proprioception

## Abstract

**Background**: Patellofemoral pain syndrome (PFPS) is a common musculoskeletal disorder that involves various biomechanical factors, including the altered positioning of the patella, weakness of the lower extremity muscles, delayed activation of the vastus medialis muscle, and excessive pronation of the foot. Although the short- and long-term effects of external support among the recommended conservative treatment methods for PFPS have been examined, there remains a lack of consensus regarding their impacts. This study was conducted to investigate the immediate effects of braces and rigid taping applied to control pain on proprioception, functional status, and balance in patients with PFPS, and to compare these outcomes with normative values obtained from healthy individuals. **Methods**: The study included 18 patients with PFPS and 18 healthy individuals who met the inclusion criteria. Through randomization of the intervention sequence, patients were evaluated under conditions of rigid taping, support, or without any support. Their pain levels before and after the application were assessed using the Visual Analog Scale; their functional status was evaluated with the Kujala Patellofemoral Scoring, the 10-Step Up Test, and the Squat; their balance performance was measured using the Y-Balance Test and the Single Leg Stance Test; and their proprioception was assessed with the Joint Position Sense Test. **Results**: It has been determined that rigid taping and bracing have similar effects in the immediate management of pain, proprioception, functional status, and balance issues in patients with PFPS. The interventions were observed to bring patients’ static balance and proprioception parameters closer to the values seen in healthy individuals. **Conclusions**: Rigid taping and bracing are both effective interventions in the management of PFPS, offering benefits such as pain relief, prevention of proprioceptive deficits, mitigation of balance impairments, and enhancement of functional outcomes. The selection of the most appropriate modality should be based on the individual patient’s characteristics and tolerance levels.

## 1. Introduction

Patellofemoral pain syndrome (PFPS) is a common condition that accounts for 25–40% of all knee disorders, with an annual prevalence of 22.7%. Characterized by anterior knee pain and/or increased pain in the retropatellar and/or peripatellar regions following flexion-related activities such as squatting, stair climbing, and prolonged sitting that increase patellofemoral compressive forces [1,2]. Despite its high prevalence, PFPS lacks a universally accepted definition, and its pathology and underlying mechanisms remain incompletely understood. The pathophysiology of the condition is considered multifactorial and has been increasingly attributed to inappropriate training loads and high-demand or periodic physical activities, in which anatomical and mechanical factors directly influence patellofemoral joint function. Lower extremity alignment and patellar instability are thought to play a significant role in symptom development, as alterations in joint kinematics may increase patellofemoral stress. Several mechanisms have been proposed, including muscular imbalances affecting lower extremity biomechanics, patellar malposition leading to articular facet compression, impaired proprioception, and soft tissue inflammation contributing to pain and dysfunction [3,4].

Several therapy procedures are proposed for researchers to use for determining the underlying causes of PFPS and treating the subsequent issues and activity limitations [5]. Conservative treatment of PFPS includes patellar taping, stretching exercises for shortened structures in the soft tissue, vastus medialis obliqus strengthening, activity modification, biofeedback, neuromuscular electrical stimulation, ultrasound, braces, and foot orthoses [6]. While the most commonly used treatment method for PFPS treatment and with a high magnitude of effect is physiotherapy and exercise training, knee-foot orthoses (braces, insoles), kinesiotape, and rigid braces are used as external supports combined with the treatment to control pain in the short and medium term tape is used [6,7]. The purpose of these supports is to correct the distorted lateral position of the patella in PFPS, and to provide correct biomechanical alignment [8]. It is known that the most frequently recommended external support for patients in interventions for PFPS in the clinical setting is kinesiology taping and bracing [9]. However, there has been no consensus as to whether patients should receive any external support during their painful periods, and the effects of these supports on pain, balance, walking, proprioception, and functionality in patients have been to be contradictory [10]. It has been stated that the use of taping and bracing in the treatment of patellofemoral pain is effective in managing pain and improving the functional levels of people, but their mechanisms of action have not yet been distinguished [11]. However, kinesiological taping does not exhibit orthotic properties such as a brace, so comparisons and studies of their effects yield misleading results [12]. The use of McConnell taping, which can produce effects similar to kinesiological taping and brace, has given clinically positive results [13].

The existing literature revealed a lack of studies that compare the efficacy of rigid taping and bracing, particularly in healthy individuals. This study aimed to investigate the immediate effects of rigid taping and a patella-stabilizing brace on pain, proprioception, function, and balance in PFPS and compare results with healthy normative values. Our hypothesis posits that rigid taping will be more effective than patella-stabilizing braces in improving proprioception, functionality, and balance in individuals with PFPS. Based on the outcomes, the study will provide recommendations for an external support approach that could enhance rehabilitation programs, ultimately improving pain management, functional performance, and balance in patients with PFPS.

## 2. Materials and Methods

### 2.1. Design

This study is a prospective, randomized controlled trial. Volunteers diagnosed with PFPS, who met the inclusion criteria and were referred to by an orthopedic surgeon for physiotherapy assessment, were included in the study. The study was conducted in accordance with the ethical principles of the Declaration of Helsinki. Before the commencement of the study, informed consent was obtained from the participants via a Volunteer Information Form. The study was approved by the Istanbul Medical Faculty Clinical Research Ethics Committee (Approval Number: 2023/105; Approval Date: 20 January 2023). The study protocol was registered in the clinical trials database (NCT05629286) on 17 November 2022.

### 2.2. Participants

Patients were sent from a university hospital and evaluated from October 2023 to July 2024 at the physiotherapy and rehabilitation unit in Istanbul University-Cerrahpasa, Faculty of Health Sciences. Forty-three volunteers, who met the inclusion criteria and were diagnosed by an orthopedic surgeon specializing in knee pathologies, were included in the study.

**Figure 1:**
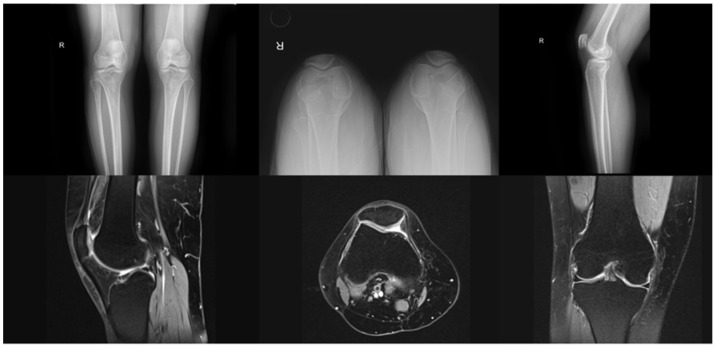
Radiological exam of Patellofemoral Pain Syndrome (PFPS).

Participants included in the study were aged between 18 and 50 years, diagnosed with PFPS, had a body mass index of less than 30 kg/m^2^, and had experienced anterior knee pain during stair climbing, squatting, and functional activities for the past three months, rating this pain as 3 or more on the visual analog scale. Individuals were excluded from the study if they had an organic lesion that could cause anterior knee pain (such as chondromalacia patellae, excessive lateral pressure syndrome, peripatellar bursitis, benign or malignant neoplasm, or tendinitis) as determined by radiological exam or MRI (Figure 1), had received an intra-articular steroid injection in the knee within the last six months, or had undergone a physiotherapy program targeting the knee joint. Additional exclusion criteria included a history of any lower extremity surgery or trauma, a diagnosis of Grade 2 or higher osteoarthritis according to the Kellgren–Lawrence scale, patellar tendinopathy, neurological issues affecting balance and walking, any rheumatologic disease, or the use of an assistive device for ambulation.

A healthy control group was included in the study to obtain normative data and to allow comparison with individuals diagnosed with PFPS. Healthy participants were recruited with consideration given to achieving similarity to the patient group in key sociodemographic characteristics, such as age and body mass index. Inclusion criteria for the healthy control group were voluntary participation, absence of any current or previous diagnosis related to the lower extremities, and no history of lower extremity injury, surgery, or musculoskeletal pain affecting daily activities. The inclusion of this group enabled the identification of deviations from normal functional and clinical parameters associated with PFPS.

### 2.3. Sample Size

To estimate the appropriate sample size, we conducted a power analysis before the study. An alpha level of *p* = 0.05 was set a priori, and power was set at 80%. The effect size was estimated at 0.4, which was calculated based on the previous literature [14]. The results of a power analysis in G*Power (version 3.1.9) indicated that 18 participants per group would provide sufficient power. Based on this information, 36 participants who met the inclusion criteria and agreed to participate in the study were divided into two groups. The intervention group consisted of 18 individuals with PFPS, while the control group included 18 healthy individuals.

### 2.4. Randomization

Randomization was determined prior to participant inclusion using a computer-generated random sequence to ensure similar conditions for each patient due to the nature of the disease progression. Randomization was performed exclusively for the PFPS group to establish the sequence of interventions. All interventions were administered to each of the 18 PFPS patients in accordance with the determined randomization order. The participants underwent evaluations in the following sequences: three patients received brace–rigid taping–no external support; three received brace–no external support–rigid taping; three received rigid taping–brace–no external support; three received rigid taping–no external support–brace; three received no external support–brace–rigid taping; and three received no external support–rigid taping–brace.

### 2.5. Procedure

The sociodemographic and clinical characteristics of the participants were recorded in individual case files. Clinical evaluations, previously standardized, were performed simultaneously by a physiotherapist. As part of the assessments, individuals with PFPS and healthy participants were assessed by the same physiotherapists. The assessments of individuals included in the healthy control group were conducted only once without any intervention. The recorded values were used to compare the effectiveness of different interventions in individuals with PFPS.

The study included three interventions designed for patients with PFPS: patella-stabilizing brace application (Ottobock, Duderstadt, Germany), rigid taping (BiaTape, İstanbul, Türkiye), and no external support. Each patient underwent all three interventions with a one-week interval between each [15]. This interval was implemented solely to ensure randomization and to avoid immediate sequential application of the interventions; outcome measures were obtained immediately after each intervention to evaluate acute effects, and no carry-over effects were assumed. During the clinical evaluations, the rigid taping and braces remained on the participants’ knee joints.

**Figure 2:**
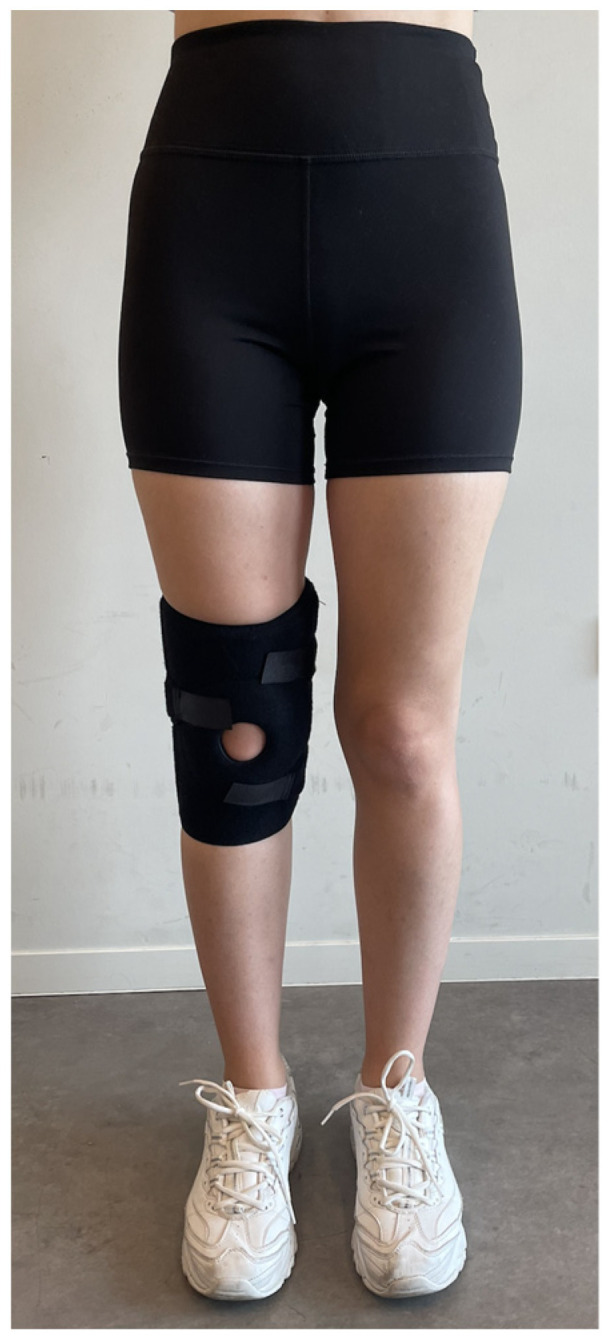
Patella-Stabilizing Brace Application.

**Figure 3:**
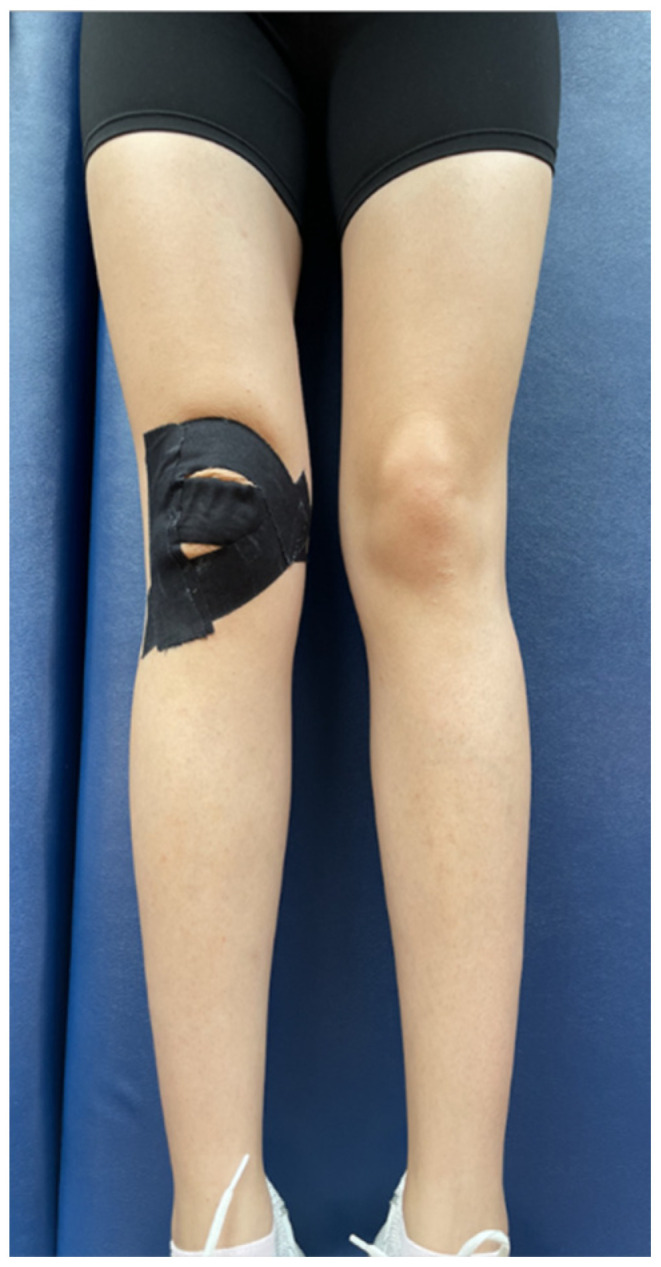
Rigid Taping Application.

To ensure that patients adapted to the external supports, they were asked not to remove the external supports in the clinic for one hour after the interventions. For the brace application, a patella-stabilizing brace (Ottobock, Duderstadt, Germany) with proven effectiveness in the literature, was used (Figure 2) [16]. The chosen brace had a patellar cutout and supported the patellofemoral joint with velcro straps for stabilization. Each participant used the same type and brand of brace assigned to them, and evaluations were conducted one hour after the brace was applied. The taping (BiaTape, İstanbul, Türkiye) procedures were carried out following McConnell’s sequence of anterior tilt, medial tilt, glide, and fat pad unloading, aiming to reduce participants’ pain by 50%. Participants were taped in a supine position with their knees at 90 degrees of flexion, using the ligament technique for the patellar tendon (100% stretch) transversely without any stretch, ensuring the knee joint remained in the midline. The taping was applied to the medial and lateral sides of the knee joint with 100% stretch, finishing above and below the patella (Figure 3) [13]. For the McConnell taping technique, participants’ knees were supported in extension while the patella was manually pushed medially into the trochlea by the physiotherapist before applying the tape. To protect the skin, a soft, hypoallergenic tape (Fixomull, Beiersdorf Australia, North Ryde, Australia) was used, and for the correction, a rigid zinc oxide tape (Endura-tape, Endura-Tape Pty Ltd., Sydney, Australia) was applied. Eligibility assessments and randomizations of participants were conducted by different researchers. The brace and rigid taping applications were administered by a physiotherapist different from those conducting the evaluations. Each participant received a single taping session and was evaluated one hour after the application.

### 2.6. Outcome Measures

The outcome assessments were performed by evaluators who were blinded to the participants’ diagnostic status (PFPS or healthy control). Due to the nature of the interventions, blinding to the presence of external support (brace or rigid taping) was not feasible; however, evaluators had no access to clinical information regarding participants’ diagnosis. For the intervention groups, a total of three assessments were performed with a one-week interval between each session, while a single measurement was conducted for the control groups. The sociodemographic and clinical characteristics of the patients were collected.

#### 2.6.1. Primary Outcome Measurements

The Visual Analog Scale (VAS), a 100 mm pain assessment scale, was marked by the patient to indicate the severity of average pain. Before the assessment, patients were instructed that the left end represented no pain, and the right end represented the most severe pain imaginable [17]. The VAS is a valid and reliable measure of pain, with a reported minimal clinically important difference of 2.0 points [18].

The functional assessment of knee complaints related to the patellofemoral structure was conducted using the Kujala Patellofemoral Score that range from 0 to 100, where higher scores indicate better outcomes [15].

#### 2.6.2. Secondary Outcome Measurements

The participants’ anterior, posterolateral, and posteromedial reach distances were evaluated using the Y-Balance Test (YBT), is a dynamic performance test used to assess dynamic postural control, lower extremity strength, flexibility, and proprioception, and is commonly employed to evaluate functional performance and injury risk of the lower extremity. During the test, participants maintained balance on the ipsilateral limb while reaching as far as possible with the contralateral limb in three directions: anterior, posteromedial, and posterolateral. The test requires simultaneous neuromuscular control and balance, making it a sensitive tool for detecting functional impairments in individuals with PFPS. The test was scored by calculating the absolute reach distance [(Reach 1 + Reach 2 + Reach 3)/3] [19]. Another functional assessment used was the 10-Step Up Test (10-SUT), which involved stepping up and down a 20 cm high step ten times [20]. To gain insights into lower extremity muscle strength and to evaluate activities that aggravate symptoms, the five-repetition double-leg squat test was employed.

To assess standing balance, the Single Leg Stance Test (SLST) was used, starting when the non-evaluated extremity lost ground contact and ending when it touched the ground again or balance was significantly disturbed [21].

Also, joint reproduction tests were used to evaluate knee joint position sense. During the evaluation, an electronic goniometer (Halo, Sydney, Australia) was used. The pivot point of the electronic goniometer was placed on the lateral condyle of the femur, with the movable arm following the fibula. After the passive positioning of the knee joint, participants were asked to actively replicate the position, and the difference from the reference angle was calculated [22].

### 2.7. Statistical Analysis

The statistical analysis of the data will be performed using the SPSS (Statistical Package for Social Sciences) 26.0 software. The normality of data distribution was assessed using the “Shapiro Wilk Test”. The statistically analyzed variables were described using mean (M) and standard deviation (SD) values. In the analysis of the study data, descriptive statistical methods (mean, standard deviation, and confidence intervals) were utilized. The Independent Sample *t*-test was applied to compare parameters with normal distribution between the intervention group, consisting of PFPS patients, and the control group of healthy individuals. For the comparison of categorical data, the Chi-square test was used. To evaluate the primary and secondary outcome measures among different interventions (brace, rigid tape, without external support) and to determine differences between these interventions, a One-Way ANOVA was conducted. The Independent Sample *t*-test was employed to compare PFPS patients’ results after each intervention with those of healthy individuals. The performance outcomes of healthy participants were considered normative values, and the percentage deviations in PFPS patients’ values from these normative values were calculated. Percentage deviation was calculated by the formula of “(calculated mean value-normative mean value)/normative mean value × 100” [22]. A *p*-value of less than 0.05 was considered statistically significant for all analyses.

## 3. Results

**Figure 4:**
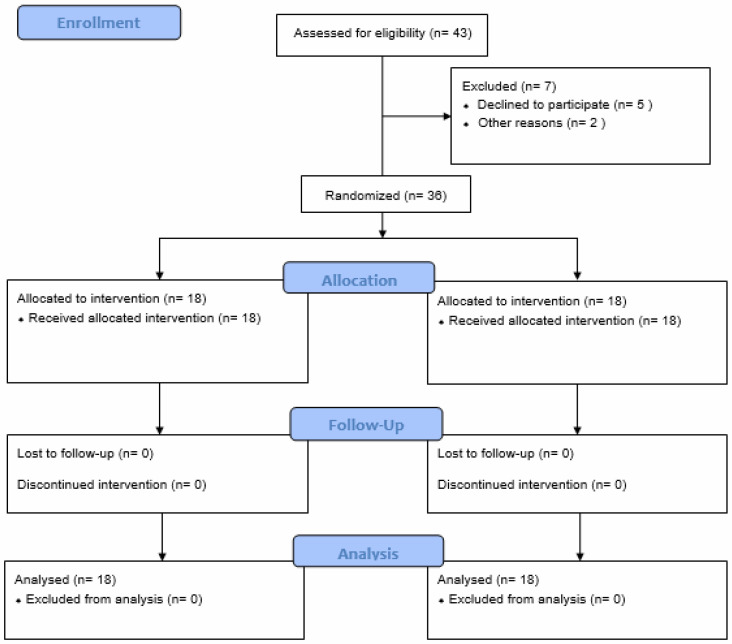
Flow Diagram.

A total of 25 patients with PFPS and 18 healthy controls who met the inclusion criteria were evaluated for the study. Five of the evaluated PFPS patients decided not to continue with the study, one withdrew due to transportation difficulties, and one participant was excluded due to an additional health issue. The study was completed with 18 PFPS patients and 18 healthy individuals (Figure 4).

### 3.1. Descriptive and Clinical Characteristics

Table 1 presents descriptive statistics for all study variables. The assessments were administered to all participants. When the descriptive and clinical characteristics of the groups were evaluated, there was no significant statistical difference between the groups.

### 3.2. The Comparison and Percentage Deviation of Interventions from Healthy Control

There were no significant statistical differences (*p* > 0.05) among the different intervention approaches regarding their effects on functional status, balance, and proprioception parameters in patients with PFPS (Table 2).

Since we could not find a study in the literature that compares the effects of bracing or rigid taping on functional status, balance, and proprioception in PFPS patients with healthy subjects, the results of the study were interpreted using normative values calculated from healthy participants included in our study.

Normative values calculated from the healthy control group and deviations from these values in PFPS patients subjected to different interventions are presented in Table 3. For the right JPT parameter, no significant statistical differences were found between healthy individuals and PFPS patients undergoing brace, rigid tape, and without external support interventions (*p* = 0.29, 0.32, and 0.12; respectively). Similarly, no significant differences were observed in the left JPT parameter with braces (*p* = 0.12) and without external support (*p* = 0.09) interventions. For the right SLST, no significant statistical differences were identified between healthy individuals and PFPS patients undergoing brace, rigid tape, and without external support interventions (0.67, 0.52, and 0.35, respectively). Likewise, no significant differences were found in the left SLST results for the same interventions (*p* = 0.57, 0.60, and 0.59; respectively). When comparing PFPS patients undergoing the rigid tape intervention with healthy individuals, no significant statistical differences were observed in the right YBT’s anterior (*p* = 0.09), posteromedial (*p* = 0.09), posterolateral (*p* = 0.16), or in the left YBT’s anterior (*p* = 0.28) and posterolateral (*p* = 0.48) parameters. Similarly, in PFPS patients evaluated with brace intervention, no significant differences were identified in the left YBT’s posteromedial (*p* = 0.13) and posterolateral (*p* = 0.07) parameters compared to healthy individuals.

## 4. Discussion

In our study, we examined the effects of tape and brace applied to patients with PFPS on their instant pain and functionality and how close they can get to the values of healthy individuals. These comparisons were intended to provide cross-sectional reference information rather than to indicate treatment-induced normalization or recovery. When comparing the immediate effects of different treatment approaches for PFPS, the results for pain, functional performance, proprioception, and balance were similar in the evaluations of bracing and rigid taping.

In the literature, we could not find any studies that compares the effects of bracing or rigid taping on functional status, balance, and proprioception in PFPS patients with those of healthy individuals. Therefore, the findings of this study were interpreted using normative values calculated from the healthy participants included in our research. However, it should be emphasized that these values represent reference data derived from the same study population and cannot fully substitute for longitudinal follow-up or pre–post comparisons within the PFPS group. Assessments conducted immediately after the interventions allowed us to examine the clinical significance of how closely the patients’ results approached those of healthy individuals. Accordingly, these findings should be interpreted as cross-sectional contrasts between groups rather than as definitive treatment effects.

Although external support applications are recommended in clinical guidelines for PFPS management, there is no consensus regarding which external support should be used [23]. We could not find a study in the literature that highlighted and compared the positioning and correction properties of rigid taping and brace applications. In these studies, the focus has been primarily on patients’ pain and functional activities, while parameters related to balance and proprioception have not been examined. In studies investigating the immediate effects of interventions on pain and functionality in PFPS, a randomized controlled trial compared the efficacy of McConnell taping and kinesiotaping during stair descent. The results have shown that McConnell taping is more effective than kinesiotaping in controlling pain and improving gait parameters during stair descent [24]. Although kinesiotaping was not applied in the current study, the corrective and stabilizing properties of rigid taping were found to be effective in influencing functional activities and pain parameters. The positive effects of rigid taping on pain parameters, along with its potential to influence knee flexion–extension angles, may have supported better performance during sit-to-stand and squat tests, which are activities that could otherwise aggravate the pathology in individuals with PFPS. In their study involving 32 individuals with PFPS, Agostini et al. [25] examined the immediate and long-term effects of rigid taping and kinesiotaping. They found that McConnell taping, applied in addition to other interventions, was superior to kinesiotaping in reducing pain and enhancing functional recovery [25]. Similarly, a systematic review analyzing 13 articles on the efficacy of patellar taping demonstrated positive effects of taping methods on pain. However, no significant improvements were observed in functionality, muscle activity, or biomechanical parameters [26]. Another study assessed the immediate effects of patellar-supportive bracing on pain and neuromuscular activity. It found significant reductions in pain scores during all activities when the brace was worn [27]. Within the scope of this study, it was not possible to conduct pre- and post-intervention comparisons of patients’ pain and functionality due to the randomized sequence of all interventions. Therefore, the outcomes obtained reflect instantaneous responses associated with each intervention rather than changes relative to a baseline condition. However, based on the obtained results, the measurements provided instantaneous outcomes, regardless of the specific intervention applied, demonstrating similarities across the interventions. Similarly, outcome measures assessing functional status revealed comparable results among all three interventions. The present study utilized rigid taping and bracing for pain management and correction, which demonstrated comparable outcomes in pain, functional performance, proprioception, and balance parameters. By randomizing the interventions for each patient, the study accounted for disease severity, ensuring a more homogeneous procedure. The well-designed methodology showed that patients receiving either intervention exhibited similar improvements. Unlike many studies in the literature, the preference for rigid taping over kinesiotaping in this study, due to its demonstrated superiority over placebo interventions in terms of pain and functional status, may have contributed to the similar outcomes observed across the different interventions [28,29]. These findings, supported by the literature, suggest that rigid taping and brace applications can effectively reduce complaints in the short term for PFPS patients.

In our study, no significant differences were observed between the application of rigid taping and bracing in terms of immediate pain control and reduction. Although we hypothesized that rigid taping would be superior to patella-stabilizing braces in improving proprioception, functionality, and balance in individuals with PFPS, our results did not support this assumption. The comparable effects observed between the two external support approaches are consistent with previous research suggesting that both taping and bracing can provide short-term benefits for pain relief and functional outcomes in PFPS, while not differing substantially in their acute effects on proprioceptive or balance-related measures. The existing literature further indicates that the effects of taping techniques on pain, proprioception, and motor function remain variable and may be modest, influenced by contextual and methodological factors rather than demonstrating consistent superiority of one intervention over another [30]. Moreover, heterogeneity in application techniques, outcome measures, and study designs has been highlighted as a limiting factor in detecting clear superiority between taping and bracing approaches in controlled comparisons [28]. Functional test outcomes, including the 10-SUT and squat test, revealed no superiority between the intervention methods, and patients with PFPS did not reach the normative values typically observed in healthy individuals following either intervention. This finding further supports the notion that reference values obtained from healthy individuals should be interpreted cautiously in the absence of longitudinal comparisons. This may be attributed to the inclusion of deep knee flexion movements, which are known to aggravate patellofemoral pain. While short-term interventions alone are not expected to substantially alter disease severity, both bracing and rigid taping resulted in better outcomes than no intervention, supporting the clinical value of incorporating external supports into routine management. Differences observed in joint position sense, particularly in the non-dominant extremities, may reflect lower proprioceptive development compared to the dominant side, whereas the clinically comparable results observed with bracing in some measures may be explained by the increased joint stability provided through motion restriction. Finally, while static balance outcomes approached those of healthy individuals, dynamic balance remained impaired, suggesting that external supports alone may be insufficient under dynamic conditions and highlighting the importance of interventions that promote active neuromuscular control. This discrepancy also indicates that cross-sectional proximity to reference values does not necessarily imply restoration of dynamic function.

In addition to the objective outcome measures, patients’ subjective responses to the interventions provided noteworthy clinical insights. Several participants reported greater perceived pain relief with the patella-stabilizing brace, whereas rigid taping was more commonly preferred in terms of functional performance during activities. Although these preferences were not formally quantified, they reflect real-world clinical decision-making, where treatment selection is often guided not only by measurable outcomes but also by patient comfort, perceived benefit, and functional confidence. Highlighting these observations may support a more individualized approach to the management of PFPS, particularly when short-term symptom relief and functional demands differ between patients.

Our study has several strengths. It examines interventions commonly used as first-line treatments for patients with PFPS, a condition frequently seen in clinics, before progressing to more extensive conservative treatments. As a result of the applied interventions, the calculation of the percentage deviations between the normal values by comparing the evaluation results with the evaluation results of healthy individuals has guided which applications should be selected in terms of clinical practice. Several limitations should be considered when interpreting the findings of this study. Although an a priori power analysis suggested that the sample size was adequate, the relatively small number of participants with PFPS represents a limitation. This sample size may have reduced the ability to detect subtle differences between interventions with inherently similar mechanisms, such as rigid taping and patella-stabilizing bracing. Therefore, the absence of observable differences between interventions should be interpreted with caution, as it may reflect either true equivalence or limited sensitivity to identify small but clinically meaningful distinctions. Additionally, the use of external supports, including braces and rigid taping, may have restricted comfortable knee movement during certain assessments, particularly those evaluating functional performance and joint position sense. Radiological assessments were not performed because the study focused on immediate functional outcomes, and the inclusion of asymptomatic controls made imaging without clinical indication ethically inappropriate. Moreover, structural alignment parameters are unlikely to explain the immediate functional changes observed following external support interventions. Future studies may incorporate imaging to explore the relationship between structural alignment and functional outcomes in PFPS.

## 5. Conclusions

The use of any external support alone without exercise therapy is not sufficient to provide positive effects on the functional status, balance, and walking of individuals in the treatment of PFPS. Among the external support options evaluated, rigid taping appeared to be associated with fewer movement restrictions compared with other applications and may therefore represent a more acceptable option in certain patients. Knee bracing may be considered as an alternative for individuals in whom rigid taping is not expected to provide adequate patellar realignment.

## Figures and Tables

**Table 1:** Descriptive and clinical characteristics of the participants.

Subjects	PFPS	Healthy Control	*p* Value
	Mean ± SD (95% CI)	Mean ± SD (95% CI)	
Gender (F/M)	12/6	15/3	0.15
Age (years)	28.50 ± 7.68 (26.39–30.56)	26.61 ± 9.79 (22.46–31.55)	0.43
Body Mass Index (kg/m^2^)	24.24 ± 3.42 (23.37–25.24)	22.90 ± 2.97 (21.54–24.36)	0.11
Dominant Side (R/L)	17/1	18/0	0.57

PFPS: patellofemoral pain syndrome; F: female; M: male; kg/m^2^: kilogram/meter square; R: right; L: left; SD: standard deviation; CI: confidence interval.

**Table 2:** The comparison of different interventions.

		Brace		Rigid Taping		Without External Support		*p* Value
		Mean ± SD	95% CI	Mean ± SD	95% CI	Mean ± SD	95% CI	
Kujala		75.72 ± 20.30	65.84–84.10	76.88 ± 18.52	67.46–84.33	75.33 ± 18.72	65.75–83.00	0.97
VAS		5.94 ± 1.58	5.22–6.58	5.66 ± 1.68	4.87–6.50	5.88 ± 1.84	5.05–6.69	0.60
10-SUT		27.23 ± 20.09	18.89–36.57	27.48 ± 21.44	18.41–39.26	28.99 ± 22.95	19.52–40.84	0.84
Squat test		14.08 ± 4.47	11.98–16.28	14.02 ± 5.74	11.72–17.03	14.80 ± 6.31	12.00–18.09	0.97
JPST	R	1.00 ± 3.97	0.90–3.11	1.16 ± 5.03	1.11–3.56	2.11 ± 5.68	0.62–4.57	0.56
	L	2.72 ± 6.99	0.30–6.07	3.94 ± 4.37	2.00–6.11	1.83 ± 4.24	0.19–3.93	0.77
SLST	R	46.82 ± 47.87	27.62–72.68	43.77 ± 44.49	26.54–68.27	40.20 ± 43.47	23.80–63.91	0.93
	L	60.07 ± 47.87	41.96–85.77	58.68 ± 38.97	42.44–79.20	58.78 ± 37.82	42.07–77.11	0.97
YBT-R	Ant	73.11 ± 17.97	64.62–81.21	77.10 ± 15.22	69.53–83.97	72.21 ± 17.58	64.70–80.17	0.67
	PM	75.49 ± 21.11	66.58–85.64	78.96 ± 21.67	68.75–89.58	73.78 ± 17.12	65.60–81.56	0.83
	PL	77.84 ± 21.25	68.39–88.30	82.06 ± 21.00	71.46–91.51	74.12 ± 21.40	64.73–84.08	0.75
YBT-L	Ant	69.88 ± 19.39	61.12–78.97	79.69 ± 17.49	71.10–87.96	73.01 ± 19.44	64.34–82.07	0.21
	PM	78.01 ± 22.92	67.56–89.05	77.36 ± 18.68	67.72–85.70	75.61 ± 17.88	67.01–84.09	0.96
	PL	79.33 ± 19.24	70.79–89.19	84.16 ± 21.08	73.28–93.67	74.35 ± 21.11	64.48–84.00	0.57

VAS: Visual Analog Scale; 10-SUT: 10-Step Up Test; JPST: Joint Position Sense Test; SLST: Single Leg Stance Test; YBT: Y-Balance Test; R: right; L: left; ant: anterior; PM: posteromedial; PL: posterolateral; SD: standard deviation; CI: confidence interval.

**Table 3:** Percentage deviation of interventions from healthy controls and comparison of interventions’ and controls’.

		Healthy Controls		Percentage Deviation of Interventions from Controls’			*p* Value of Interventions from Healthy Controls’		
		Mean ± SD	95% CI	Brace	Rigid Taping	Without External Support	Brace	Rigid Taping	Without External Support
Kujala		98.50 ± 2.64	97.19–99.61	23.12	21.94	23.52	<0.001	<0.001	<0.001
VAS		0.00 ± 0.00	0.00–0.00	NA	NA	NA	<0.001	<0.001	<0.001
10-SUT		16.29 ± 2.97	14.83–17.51	67.15	68.69	77.96	0.03	0.04	0.03
Squat test		9.09 ± 2.46	8.07–10.33	54.89	54.23	62.81	<0.001	0.003	0.002
JPST	R	0.11 ± 1.77	0.76–1.00	809.09	954.54	1818.18	0.29	0.32	0.12
	L	0.05 ± 1.89	0.78–1.00	5320	7780	3560	0.12	0.002	0.09
SLST	R	52.79 ± 37.35	37.64–72.53	11.30	17.08	23.84	0.67	0.51	0.35
	L	51.86 ± 38.62	36.37–71.83	15.83	13.15	13.34	0.57	0.60	0.59
YBT-R	Ant	87.56 ± 20.71	77.88–97.71	16.50	11.94	17.53	0.03	0.09	0.02
	PM	93.67 ± 28.49	80.57–107.17	19.40	15.70	21.23	0.03	0.09	0.01
	PL	91.51 ± 18.40	82.98–99.98	14.93	10.32	19.00	0.04	0.16	0.01
YBT-L	Ant	86.43 ± 19.82	77.55–95.41	19.14	7.79	15.52	0.01	0.28	0.04
	PM	91.01 ± 18.88	82.99–99.38	14.28	14.99	16.92	0.13	0.03	0.01
	PL	88.72 ± 17.37	80.99–97.20	10.58	5.13	16.19	0.07	0.48	0.03

VAS: Visual Analog Scale; 10-SUT: 10-Step Up Test; JPST: Joint Position Sense Test; SLST: Single Leg Stance Test; YBT: Y-Balance Test; R: right; L: left; ant: anterior; PM: posteromedial; PL: posterolateral; SD: standard deviation; CI: confidence interval. Because healthy individuals did not report any pain, percentage deviation for the VAS could not be calculated; therefore, it is presented as “NA” in the table.

## Data Availability

The data presented in this study are available on request from the corresponding author.
